# Supplementary figures and images for: Deep learning reveals endogenous sterols as allosteric modulators of the GPCR–Gα interface
Source: eLife. 2025 Dec 8;14:RP106397. doi: 10.7554/eLife.106397 (PMC12685305; doi:10.7554/eLife.106397)

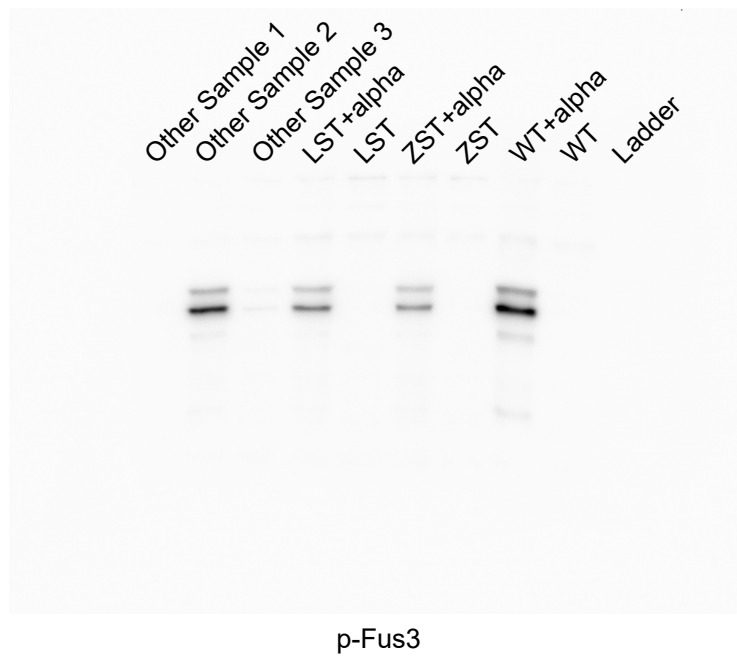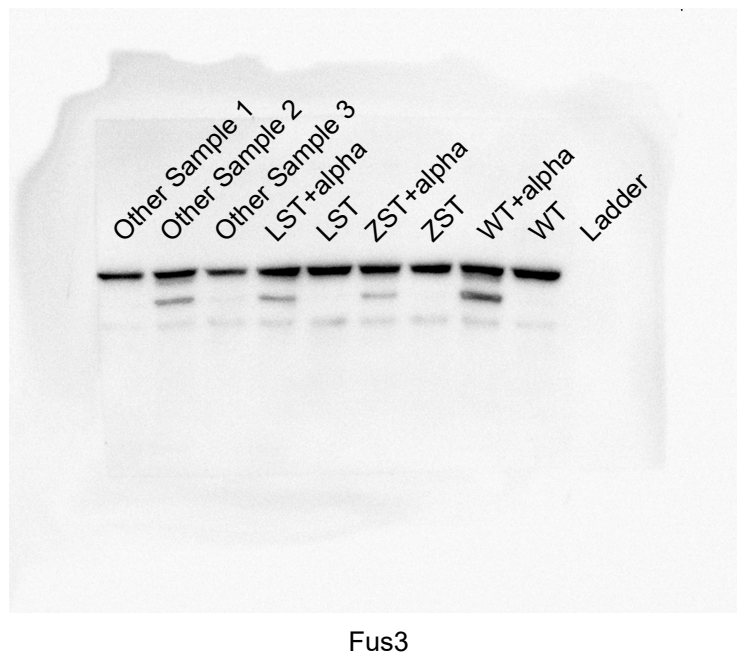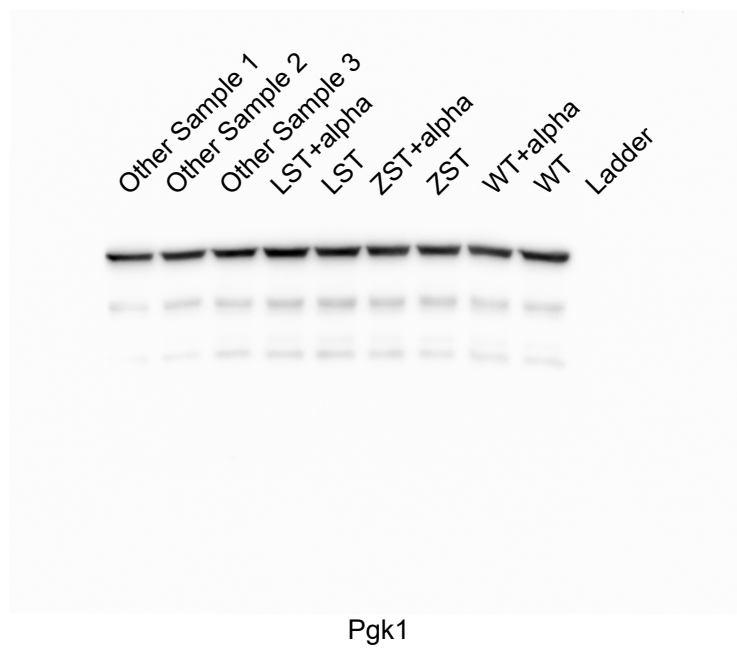

Supplement: Figure 3—source data 1. [file elife-106397-fig3-data1.pdf]

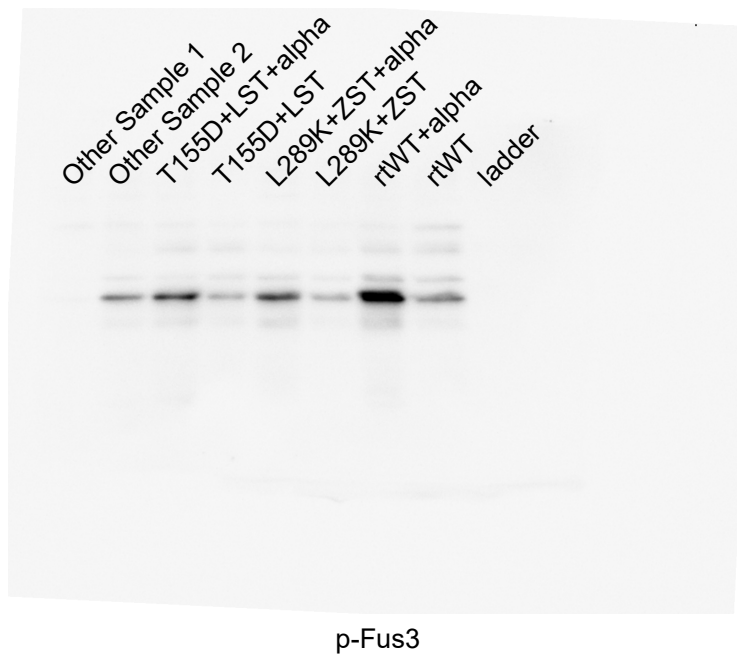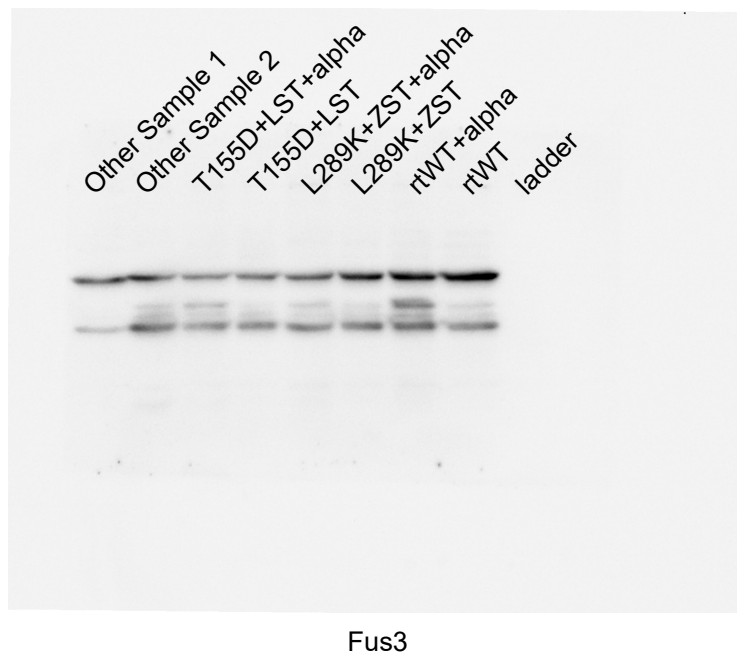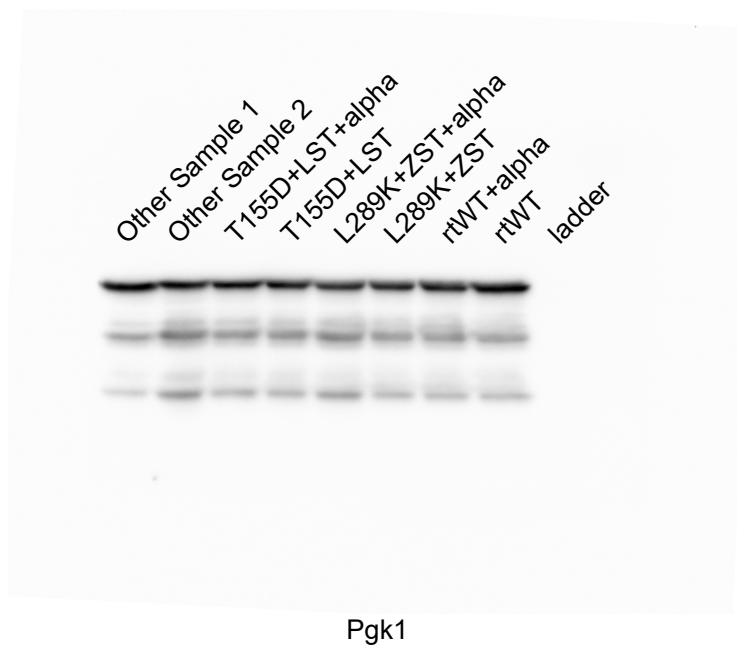

Supplement: Figure 4—source data 1. [file elife-106397-fig4-data1.pdf]
