# Supplementary material for: Deep learning reveals endogenous sterols as allosteric modulators of the GPCR–Gα interface
Source: eLife. 2025 Dec 8;14:RP106397. doi: 10.7554/eLife.106397 (PMC12685305; doi:10.7554/eLife.106397)
Supplement: Supplementary file 13. — Each row starts with the sequence identifier followed by the aligned sequence (in chunks) with the ending position of the aligned sequence provided at the end, separated by tabs. The mutation sites are highlighted in red (S75A), yellow (T155D), and blue (L289K). [file elife-106397-supp13.docx]

**Nucleotide Alignment**

STE2-S75A ATGTCTGATGCGGCTCCTTCATTGAGCAATCTATTTTATGATCCAACGTATAATCCTGGT 60

STE2-Wt ATGTCTGATGCGGCTCCTTCATTGAGCAATCTATTTTATGATCCAACGTATAATCCTGGT 60

STE2-L289K ATGTCTGATGCGGCTCCTTCATTGAGCAATCTATTTTATGATCCAACGTATAATCCTGGT 60

STE2-T155D ATGTCTGATGCGGCTCCTTCATTGAGCAATCTATTTTATGATCCAACGTATAATCCTGGT 60

STE2-S75A CAAAGCACCATTAACTACACTTCCATATATGGGAATGGATCTACCATCACTTTCGATGAG 120

STE2-Wt CAAAGCACCATTAACTACACTTCCATATATGGGAATGGATCTACCATCACTTTCGATGAG 120

STE2-L289K CAAAGCACCATTAACTACACTTCCATATATGGGAATGGATCTACCATCACTTTCGATGAG 120

STE2-T155D CAAAGCACCATTAACTACACTTCCATATATGGGAATGGATCTACCATCACTTTCGATGAG 120

STE2-S75A TTGCAAGGTTTAGTTAACAGTACTGTTACTCAGGCCATTATGTTTGGTGTCAGATGTGGT 180

STE2-Wt TTGCAAGGTTTAGTTAACAGTACTGTTACTCAGGCCATTATGTTTGGTGTCAGATGTGGT 180

STE2-L289K TTGCAAGGTTTAGTTAACAGTACTGTTACTCAGGCCATTATGTTTGGTGTCAGATGTGGT 180

STE2-T155D TTGCAAGGTTTAGTTAACAGTACTGTTACTCAGGCCATTATGTTTGGTGTCAGATGTGGT 180

STE2-S75A GCAGCTGCTTTGACTTTGATTGTCATGTGGATGACATCGAGAGCTAGAAAAACGCCGATT 240

STE2-Wt GCAGCTGCTTTGACTTTGATTGTCATGTGGATGACATCGAGAAGCAGAAAAACGCCGATT 240

STE2-L289K GCAGCTGCTTTGACTTTGATTGTCATGTGGATGACATCGAGAAGCAGAAAAACGCCGATT 240

STE2-T155D GCAGCTGCTTTGACTTTGATTGTCATGTGGATGACATCGAGAAGCAGAAAAACGCCGATT 240

STE2-S75A TTCATTATCAACCAAGTTTCATTGTTTTTAATCATTTTGCATTCTGCACTCTATTTTAAA 300

STE2-Wt TTCATTATCAACCAAGTTTCATTGTTTTTAATCATTTTGCATTCTGCACTCTATTTTAAA 300

STE2-L289K TTCATTATCAACCAAGTTTCATTGTTTTTAATCATTTTGCATTCTGCACTCTATTTTAAA 300

STE2-T155D TTCATTATCAACCAAGTTTCATTGTTTTTAATCATTTTGCATTCTGCACTCTATTTTAAA 300

STE2-S75A TATTTACTGTCTAATTACTCTTCAGTGACTTACGCTCTCACCGGATTTCCTCAGTTCATC 360

STE2-Wt TATTTACTGTCTAATTACTCTTCAGTGACTTACGCTCTCACCGGATTTCCTCAGTTCATC 360

STE2-L289K TATTTACTGTCTAATTACTCTTCAGTGACTTACGCTCTCACCGGATTTCCTCAGTTCATC 360

STE2-T155D TATTTACTGTCTAATTACTCTTCAGTGACTTACGCTCTCACCGGATTTCCTCAGTTCATC 360

STE2-S75A AGTAGAGGTGACGTTCATGTTTATGGTGCTACAAATATAATTCAAGTCCTTCTTGTGGCT 420

STE2-Wt AGTAGAGGTGACGTTCATGTTTATGGTGCTACAAATATAATTCAAGTCCTTCTTGTGGCT 420

STE2-L289K AGTAGAGGTGACGTTCATGTTTATGGTGCTACAAATATAATTCAAGTCCTTCTTGTGGCT 420

STE2-T155D AGTAGAGGTGACGTTCATGTTTATGGTGCTACAAATATAATTCAAGTCCTTCTTGTGGCT 420

STE2-S75A TCTATTGAGACTTCACTGGTGTTTCAGATAAAAGTTATTTTCACAGGCGACAACTTCAAA 480

STE2-Wt TCTATTGAGACTTCACTGGTGTTTCAGATAAAAGTTATTTTCACAGGCGACAACTTCAAA 480

STE2-L289K TCTATTGAGACTTCACTGGTGTTTCAGATAAAAGTTATTTTCACAGGCGACAACTTCAAA 480

STE2-T155D TCTATTGAGACTTCACTGGTGTTTCAGATAAAAGTTATTTTCGACGGCGACAACTTCAAA 480

STE2-S75A AGGATAGGTTTGATGCTGACGTCGATATCTTTCACTTTAGGGATTGCTACAGTTACCATG 540

STE2-Wt AGGATAGGTTTGATGCTGACGTCGATATCTTTCACTTTAGGGATTGCTACAGTTACCATG 540

STE2-L289K AGGATAGGTTTGATGCTGACGTCGATATCTTTCACTTTAGGGATTGCTACAGTTACCATG 540

STE2-T155D AGGATAGGTTTGATGCTGACGTCGATATCTTTCACTTTAGGGATTGCTACAGTTACCATG 540

STE2-S75A TATTTTGTAAGCGCTGTTAAAGGTATGATTGTGACTTATAATGATGTTAGTGCCACCCAA 600

STE2-Wt TATTTTGTAAGCGCTGTTAAAGGTATGATTGTGACTTATAATGATGTTAGTGCCACCCAA 600

STE2-L289K TATTTTGTAAGCGCTGTTAAAGGTATGATTGTGACTTATAATGATGTTAGTGCCACCCAA 600

STE2-T155D TATTTTGTAAGCGCTGTTAAAGGTATGATTGTGACTTATAATGATGTTAGTGCCACCCAA 600

STE2-S75A GATAAATACTTCAATGCATCCACAATTTTACTTGCATCCTCAATAAACTTTATGTCATTT 660

STE2-Wt GATAAATACTTCAATGCATCCACAATTTTACTTGCATCCTCAATAAACTTTATGTCATTT 660

STE2-L289K GATAAATACTTCAATGCATCCACAATTTTACTTGCATCCTCAATAAACTTTATGTCATTT 660

STE2-T155D GATAAATACTTCAATGCATCCACAATTTTACTTGCATCCTCAATAAACTTTATGTCATTT 660

STE2-S75A GTCCTGGTAGTTAAATTGATTTTAGCTATTAGATCAAGAAGATTCCTTGGTCTCAAGCAG 720

STE2-Wt GTCCTGGTAGTTAAATTGATTTTAGCTATTAGATCAAGAAGATTCCTTGGTCTCAAGCAG 720

STE2-L289K GTCCTGGTAGTTAAATTGATTTTAGCTATTAGATCAAGAAGATTCCTTGGTCTCAAGCAG 720

STE2-T155D GTCCTGGTAGTTAAATTGATTTTAGCTATTAGATCAAGAAGATTCCTTGGTCTCAAGCAG 720

STE2-S75A TTCGATAGTTTCCATATTTTACTCATAATGTCATGTCAATCTTTGTTGGTTCCATCGATA 780

STE2-Wt TTCGATAGTTTCCATATTTTACTCATAATGTCATGTCAATCTTTGTTGGTTCCATCGATA 780

STE2-L289K TTCGATAGTTTCCATATTTTACTCATAATGTCATGTCAATCTTTGTTGGTTCCATCGATA 780

STE2-T155D TTCGATAGTTTCCATATTTTACTCATAATGTCATGTCAATCTTTGTTGGTTCCATCGATA 780

STE2-S75A ATATTCATCCTCGCATACAGTTTGAAACCAAACCAGGGAACAGATGTCTTGACTACTGTT 840

STE2-Wt ATATTCATCCTCGCATACAGTTTGAAACCAAACCAGGGAACAGATGTCTTGACTACTGTT 840

STE2-L289K ATATTCATCCTCGCATACAGTTTGAAACCAAACCAGGGAACAGATGTCTTGACTACTGTT 840

STE2-T155D ATATTCATCCTCGCATACAGTTTGAAACCAAACCAGGGAACAGATGTCTTGACTACTGTT 840

STE2-S75A GCAACATTACTTGCTGTATTGTCTTTACCATTATCATCAATGTGGGCCACGGCTGCTAAT 900

STE2-Wt GCAACATTACTTGCTGTATTGTCTTTACCATTATCATCAATGTGGGCCACGGCTGCTAAT 900

STE2-L289K GCAACATTACTTGCTGTATTGTCTAAACCATTATCATCAATGTGGGCCACGGCTGCTAAT 900

STE2-T155D GCAACATTACTTGCTGTATTGTCTTTACCATTATCATCAATGTGGGCCACGGCTGCTAAT 900

STE2-S75A AATGCATCCAAAACAAACACAATTACTTCAGACTTTACAACATCCACAGATAGGTTTTAT 960

STE2-Wt AATGCATCCAAAACAAACACAATTACTTCAGACTTTACAACATCCACAGATAGGTTTTAT 960

STE2-L289K AATGCATCCAAAACAAACACAATTACTTCAGACTTTACAACATCCACAGATAGGTTTTAT 960

STE2-T155D AATGCATCCAAAACAAACACAATTACTTCAGACTTTACAACATCCACAGATAGGTTTTAT 960

STE2-S75A CCAGGCACGCTGTCTAGCTTTCAAACTGATAGTATCAACAACGATGCTAAAAGCAGTCTC 1020

STE2-Wt CCAGGCACGCTGTCTAGCTTTCAAACTGATAGTATCAACAACGATGCTAAAAGCAGTCTC 1020

STE2-L289K CCAGGCACGCTGTCTAGCTTTCAAACTGATAGTATCAACAACGATGCTAAAAGCAGTCTC 1020

STE2-T155D CCAGGCACGCTGTCTAGCTTTCAAACTGATAGTATCAACAACGATGCTAAAAGCAGTCTC 1020

STE2-S75A AGAAGTAGATTATATGACCTATATCCTAGAAGGAAGGAAACAACATCGGATAAACATTCG 1080

STE2-Wt AGAAGTAGATTATATGACCTATATCCTAGAAGGAAGGAAACAACATCGGATAAACATTCG 1080

STE2-L289K AGAAGTAGATTATATGACCTATATCCTAGAAGGAAGGAAACAACATCGGATAAACATTCG 1080

STE2-T155D AGAAGTAGATTATATGACCTATATCCTAGAAGGAAGGAAACAACATCGGATAAACATTCG 1080

STE2-S75A GAAAGAACTTTTGTTTCTGAGACTGCAGATGATATAGAGAAAAATCAGTTTTATCAGTTG 1140

STE2-Wt GAAAGAACTTTTGTTTCTGAGACTGCAGATGATATAGAGAAAAATCAGTTTTATCAGTTG 1140

STE2-L289K GAAAGAACTTTTGTTTCTGAGACTGCAGATGATATAGAGAAAAATCAGTTTTATCAGTTG 1140

STE2-T155D GAAAGAACTTTTGTTTCTGAGACTGCAGATGATATAGAGAAAAATCAGTTTTATCAGTTG 1140

STE2-S75A CCCACACCTACGAGTTCAAAAAATACTAGGATAGGACCGTTTGCTGATGCAAGTTACAAA 1200

STE2-Wt CCCACACCTACGAGTTCAAAAAATACTAGGATAGGACCGTTTGCTGATGCAAGTTACAAA 1200

STE2-L289K CCCACACCTACGAGTTCAAAAAATACTAGGATAGGACCGTTTGCTGATGCAAGTTACAAA 1200

STE2-T155D CCCACACCTACGAGTTCAAAAAATACTAGGATAGGACCGTTTGCTGATGCAAGTTACAAA 1200

STE2-S75A GAGGGAGAAGTTGAACCCGTCGACATGTACACTCCCGATACGGCAGCTGATGAGGAAGCC 1260

STE2-Wt GAGGGAGAAGTTGAACCCGTCGACATGTACACTCCCGATACGGCAGCTGATGAGGAAGCC 1260

STE2-L289K GAGGGAGAAGTTGAACCCGTCGACATGTACACTCCCGATACGGCAGCTGATGAGGAAGCC 1260

STE2-T155D GAGGGAGAAGTTGAACCCGTCGACATGTACACTCCCGATACGGCAGCTGATGAGGAAGCC 1260

STE2-S75A AGAAAGTTCTGGACTGAAGATAATAATAATTTATGA 1296

STE2-Wt AGAAAGTTCTGGACTGAAGATAATAATAATTTATGA 1296

STE2-L289K AGAAAGTTCTGGACTGAAGATAATAATAATTTATGA 1296

STE2-T155D AGAAAGTTCTGGACTGAAGATAATAATAATTTATGA 1296

**Amino acid Alignment**

STE2-T155D MSDAAPSLSNLFYDPTYNPGQSTINYTSIYGNGSTITFDELQGLVNSTVTQAIMFGVRCG 60

STE2-L289K MSDAAPSLSNLFYDPTYNPGQSTINYTSIYGNGSTITFDELQGLVNSTVTQAIMFGVRCG 60

STE2-Wt MSDAAPSLSNLFYDPTYNPGQSTINYTSIYGNGSTITFDELQGLVNSTVTQAIMFGVRCG 60

STE2-S75A MSDAAPSLSNLFYDPTYNPGQSTINYTSIYGNGSTITFDELQGLVNSTVTQAIMFGVRCG 60

STE2-T155D AAALTLIVMWMTSRSRKTPIFIINQVSLFLIILHSALYFKYLLSNYSSVTYALTGFPQFI 120

STE2-L289K AAALTLIVMWMTSRSRKTPIFIINQVSLFLIILHSALYFKYLLSNYSSVTYALTGFPQFI 120

STE2-Wt AAALTLIVMWMTSRSRKTPIFIINQVSLFLIILHSALYFKYLLSNYSSVTYALTGFPQFI 120

STE2-S75A AAALTLIVMWMTSRARKTPIFIINQVSLFLIILHSALYFKYLLSNYSSVTYALTGFPQFI 120

STE2-T155D SRGDVHVYGATNIIQVLLVASIETSLVFQIKVIFDGDNFKRIGLMLTSISFTLGIATVTM 180

STE2-L289K SRGDVHVYGATNIIQVLLVASIETSLVFQIKVIFTGDNFKRIGLMLTSISFTLGIATVTM 180

STE2-Wt SRGDVHVYGATNIIQVLLVASIETSLVFQIKVIFTGDNFKRIGLMLTSISFTLGIATVTM 180

STE2-S75A SRGDVHVYGATNIIQVLLVASIETSLVFQIKVIFTGDNFKRIGLMLTSISFTLGIATVTM 180

STE2-T155D YFVSAVKGMIVTYNDVSATQDKYFNASTILLASSINFMSFVLVVKLILAIRSRRFLGLKQ 240

STE2-L289K YFVSAVKGMIVTYNDVSATQDKYFNASTILLASSINFMSFVLVVKLILAIRSRRFLGLKQ 240

STE2-Wt YFVSAVKGMIVTYNDVSATQDKYFNASTILLASSINFMSFVLVVKLILAIRSRRFLGLKQ 240

STE2-S75A YFVSAVKGMIVTYNDVSATQDKYFNASTILLASSINFMSFVLVVKLILAIRSRRFLGLKQ 240

STE2-T155D FDSFHILLIMSCQSLLVPSIIFILAYSLKPNQGTDVLTTVATLLAVLSLPLSSMWATAAN 300

STE2-L289K FDSFHILLIMSCQSLLVPSIIFILAYSLKPNQGTDVLTTVATLLAVLSKPLSSMWATAAN 300

STE2-Wt FDSFHILLIMSCQSLLVPSIIFILAYSLKPNQGTDVLTTVATLLAVLSLPLSSMWATAAN 300

STE2-S75A FDSFHILLIMSCQSLLVPSIIFILAYSLKPNQGTDVLTTVATLLAVLSLPLSSMWATAAN 300

STE2-T155D NASKTNTITSDFTTSTDRFYPGTLSSFQTDSINNDAKSSLRSRLYDLYPRRKETTSDKHS 360

STE2-L289K NASKTNTITSDFTTSTDRFYPGTLSSFQTDSINNDAKSSLRSRLYDLYPRRKETTSDKHS 360

STE2-Wt NASKTNTITSDFTTSTDRFYPGTLSSFQTDSINNDAKSSLRSRLYDLYPRRKETTSDKHS 360

STE2-S75A NASKTNTITSDFTTSTDRFYPGTLSSFQTDSINNDAKSSLRSRLYDLYPRRKETTSDKHS 360

STE2-T155D ERTFVSETADDIEKNQFYQLPTPTSSKNTRIGPFADASYKEGEVEPVDMYTPDTAADEEA 420

STE2-L289K ERTFVSETADDIEKNQFYQLPTPTSSKNTRIGPFADASYKEGEVEPVDMYTPDTAADEEA 420

STE2-Wt ERTFVSETADDIEKNQFYQLPTPTSSKNTRIGPFADASYKEGEVEPVDMYTPDTAADEEA 420

STE2-S75A ERTFVSETADDIEKNQFYQLPTPTSSKNTRIGPFADASYKEGEVEPVDMYTPDTAADEEA 420

STE2-T155D RKFWTEDNNNL 431

STE2-L289K RKFWTEDNNNL 431

STE2-Wt RKFWTEDNNNL 431

STE2-S75A RKFWTEDNNNL 431
